# Supplementary material for: The Pentameric Ligand-Gated Ion Channel Family: A New Member of the Voltage Gated Ion Channel Superfamily?
Source: Int J Mol Sci. 2024 May 3;25(9):5005. doi: 10.3390/ijms25095005 (PMC11084639; doi:10.3390/ijms25095005)
Supplement: Supplementary file 1 [file ijms-25-05005-s001.zip › Figure_S1.pdf]

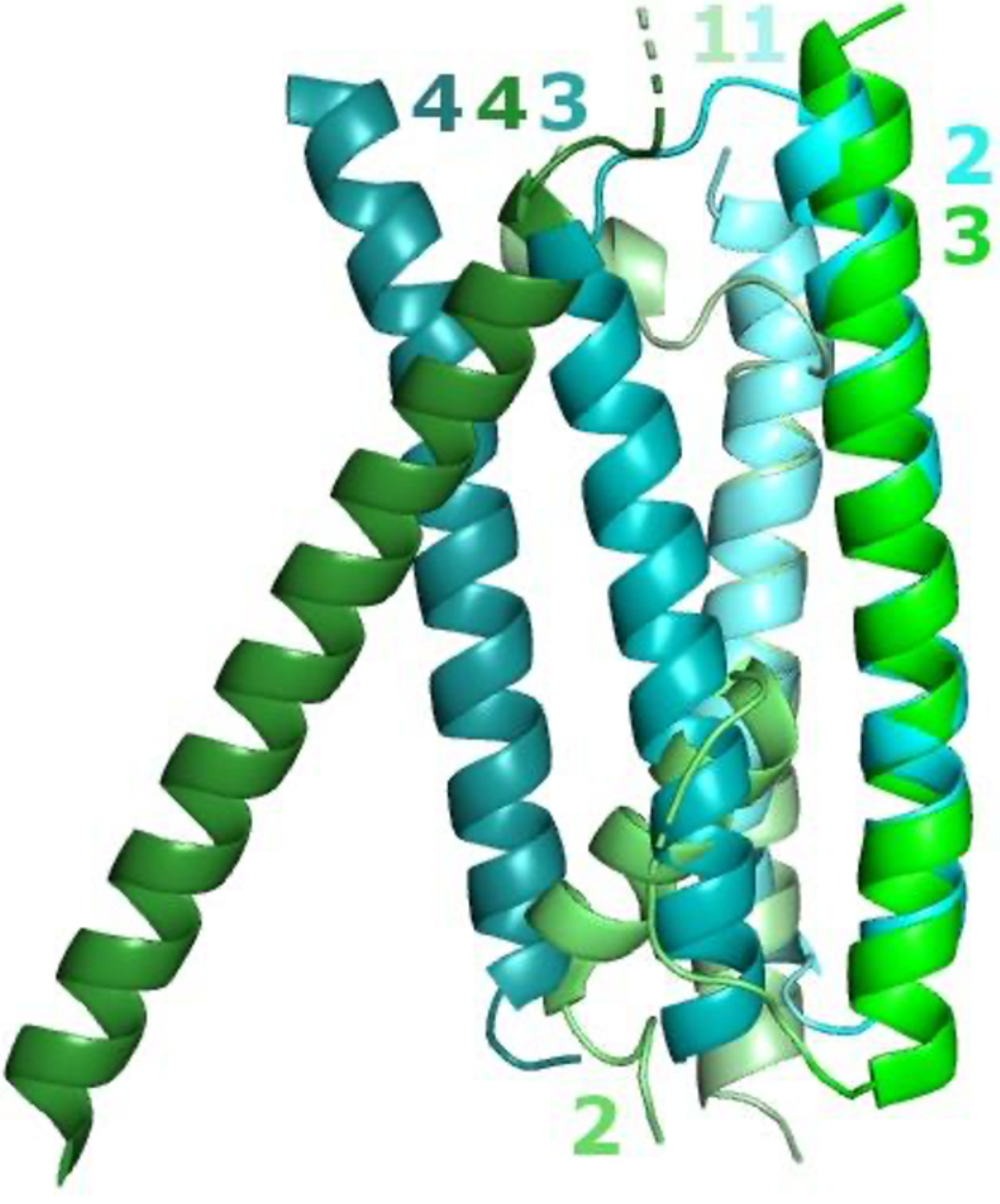

Figure S1. Structural Alignment between families pLIC and GIC. 3D structural superposition of transmembrane regions between pLIC member nicotinic acetylcholine receptor δ subunit (CHRND; 1OED; cyan) and GIC member Glutamate receptor 1 (Gria1; 6NJN; green). Transmembrane α-helices 1-4 for 1OED, chain C, and 6NJN, chain A, are labeled and shaded from light to dark in the order they appear in the amino acid sequence. TMSs 1 align between both structures, TMS 2 of 1OED aligns with TMS 3 of 6NJN. The rest of the helices in both structures could not be aligned due to their inverted orientations caused by the reentrant loop (helix 2) in the GIC structure. The scores of the alignment are RMSD: 4.06 Å, TM-align: 0.42547, and coverage: 61.42%. Note that both structures have poor resolutions (> 4.0 Å)
