# Supplementary material for: The Pentameric Ligand-Gated Ion Channel Family: A New Member of the Voltage Gated Ion Channel Superfamily?
Source: Int J Mol Sci. 2024 May 3;25(9):5005. doi: 10.3390/ijms25095005 (PMC11084639; doi:10.3390/ijms25095005)
Supplement: Supplementary file 1 [file ijms-25-05005-s001.zip › Figure_S2.pdf]

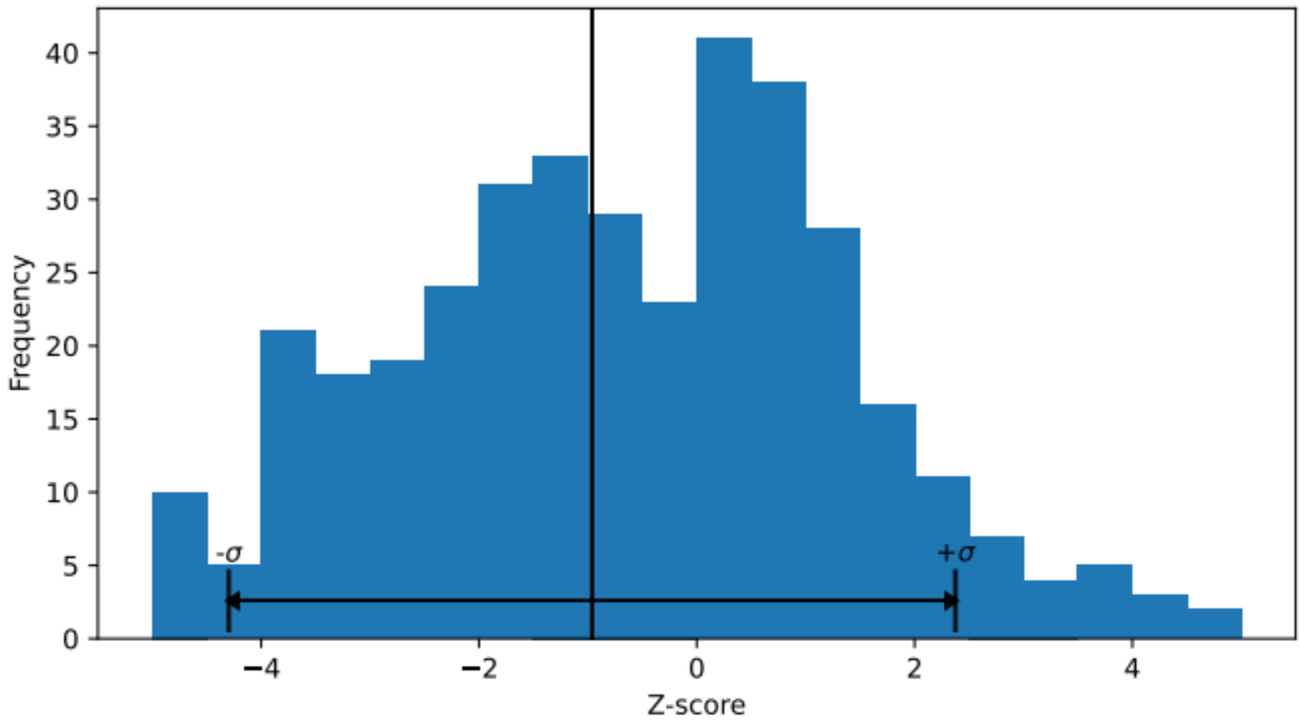

Figure S2. Differences between same-protein reentrant loop lengths and mean TMS lengths. This histogram shows Z-scores for comparisons between reentrant loop (RL) lengths and mean TMS lengths based on 412 reentrant loops in 262 member proteins of the VIC superfamily with at least one reentrant loop and at least two TMSs. To account for potential biases in the width of the membrane planes and the lengths of TMSs and RLs inferred by TmAlphaFold, we estimated a normalized Z-score per RL i in protein j ($Z_{i,j}$), based on the length of RL i ($l_{i,j}$) and the mean (μ_j^{tms}) and standard deviation (σ_j^{tms}) of TMS lengths per protein j as:

$$Z_{i,j} = \left(l_{i,j} - \mu_j^{tms}\right)/\sigma_j^{tms},$$

where negative values indicate that the average TMS length in protein j is larger than the length of the corresponding RL i. The bimodal distribution of $Z_{i,j}$ highlights the considerable frequency with which RLs are larger than the average TMS. The vertical line marks the mean Z-score ($\mu_Z = -0.96$) of the distribution. The extents at the endpoints of the horizontal line mark out the range of $Z_{i,j}$ within one standard deviation of μ_Z ($\sigma_Z = 3.33$). With $Z_{i,j} = 0$ well within a standard deviation of μ_Z, we cannot reliably conclude that reentrant loop lengths differ significantly from the mean TMS lengths in the VIC superfamily.
