# Supplementary material for: The Pentameric Ligand-Gated Ion Channel Family: A New Member of the Voltage Gated Ion Channel Superfamily?
Source: Int J Mol Sci. 2024 May 3;25(9):5005. doi: 10.3390/ijms25095005 (PMC11084639; doi:10.3390/ijms25095005)
Supplement: Supplementary file 1 [file ijms-25-05005-s001.zip › Figure_S3.pdf]

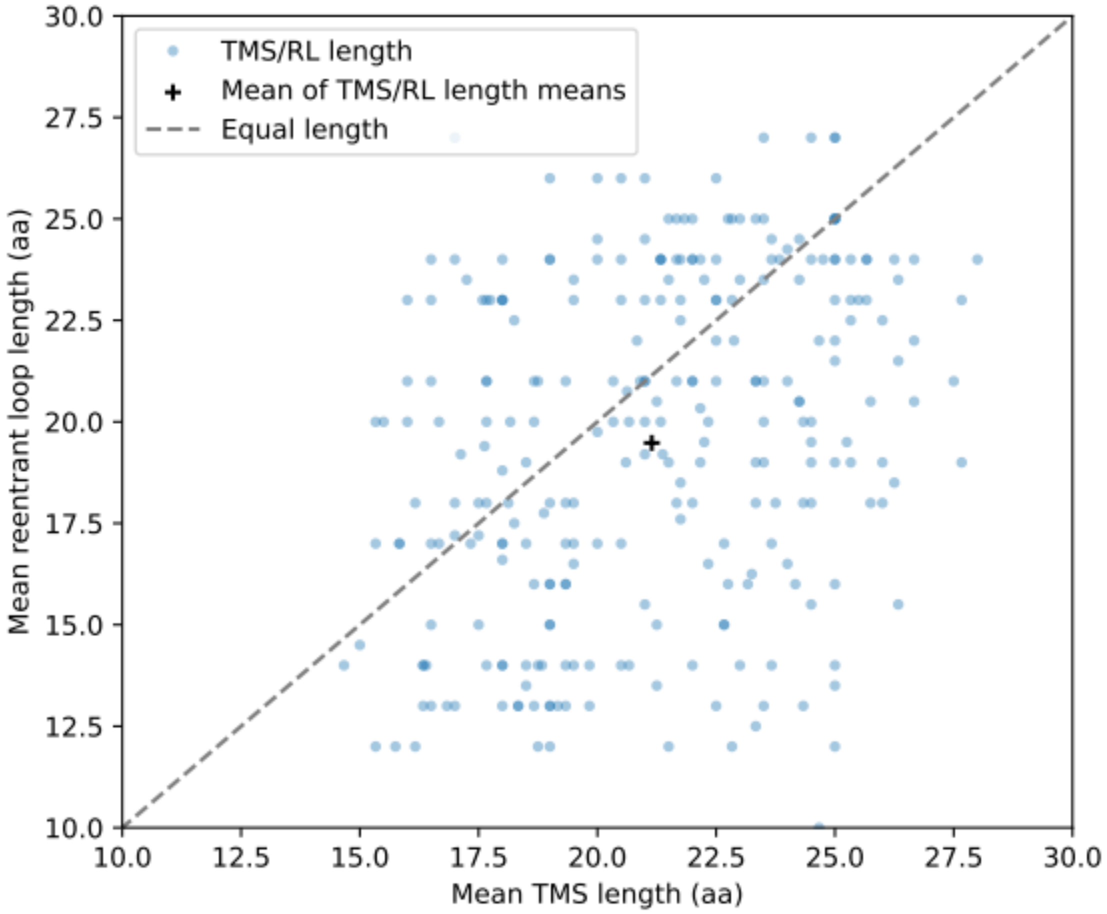

Figure S3. Scatter plot of same-protein mean TMS lengths and mean reentrant loop lengths. Plotted in this figure as blue dots are mean TMS lengths and mean reentrant loop lengths for 270 member proteins of the VIC superfamily with at least one reentrant loop and at least one TMS. There is no significant correlation between the two lengths ($r^2 = 0.10$). The black plus (+) indicates the mean TMS length and reentrant loop length, and the dashed gray line traces out the set of points where mean TMS length is equal to mean reentrant loop length.
