# Supplementary material for: The Pentameric Ligand-Gated Ion Channel Family: A New Member of the Voltage Gated Ion Channel Superfamily?
Source: Int J Mol Sci. 2024 May 3;25(9):5005. doi: 10.3390/ijms25095005 (PMC11084639; doi:10.3390/ijms25095005)
Supplement: Supplementary file 1 [file ijms-25-05005-s001.zip › Figure_S4.pdf]

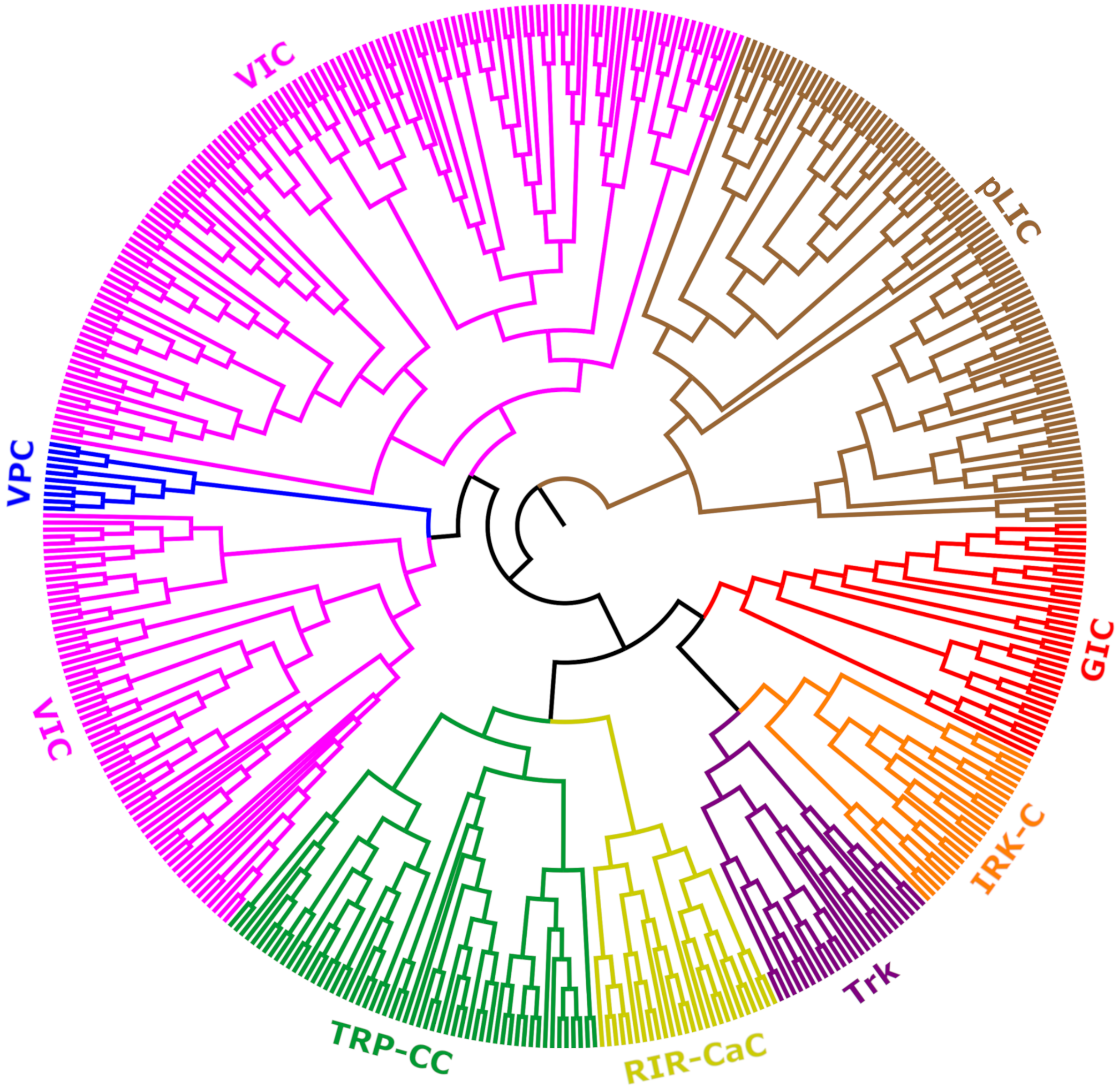

Figure S4. The VIC superfamily tree. Relationship among all 7 families within the VIC superfamily and the pLIC family. Only established members from each family in TCDB were used. Each family is labeled and shown in different colors. The R package (https://www.r-project.org/) was used to run hierarchical clustering of Smith-Waterman pairwise alignment bitscores (Ward method; agglomerative coefficient: 0.995; See Methods). Note that families GIC and VPC are the most distant families in their respective branches.
