# Supplementary material for: The Pentameric Ligand-Gated Ion Channel Family: A New Member of the Voltage Gated Ion Channel Superfamily?
Source: Int J Mol Sci. 2024 May 3;25(9):5005. doi: 10.3390/ijms25095005 (PMC11084639; doi:10.3390/ijms25095005)
Supplement: Supplementary file 1 [file ijms-25-05005-s001.zip › Figure_S5.pdf]

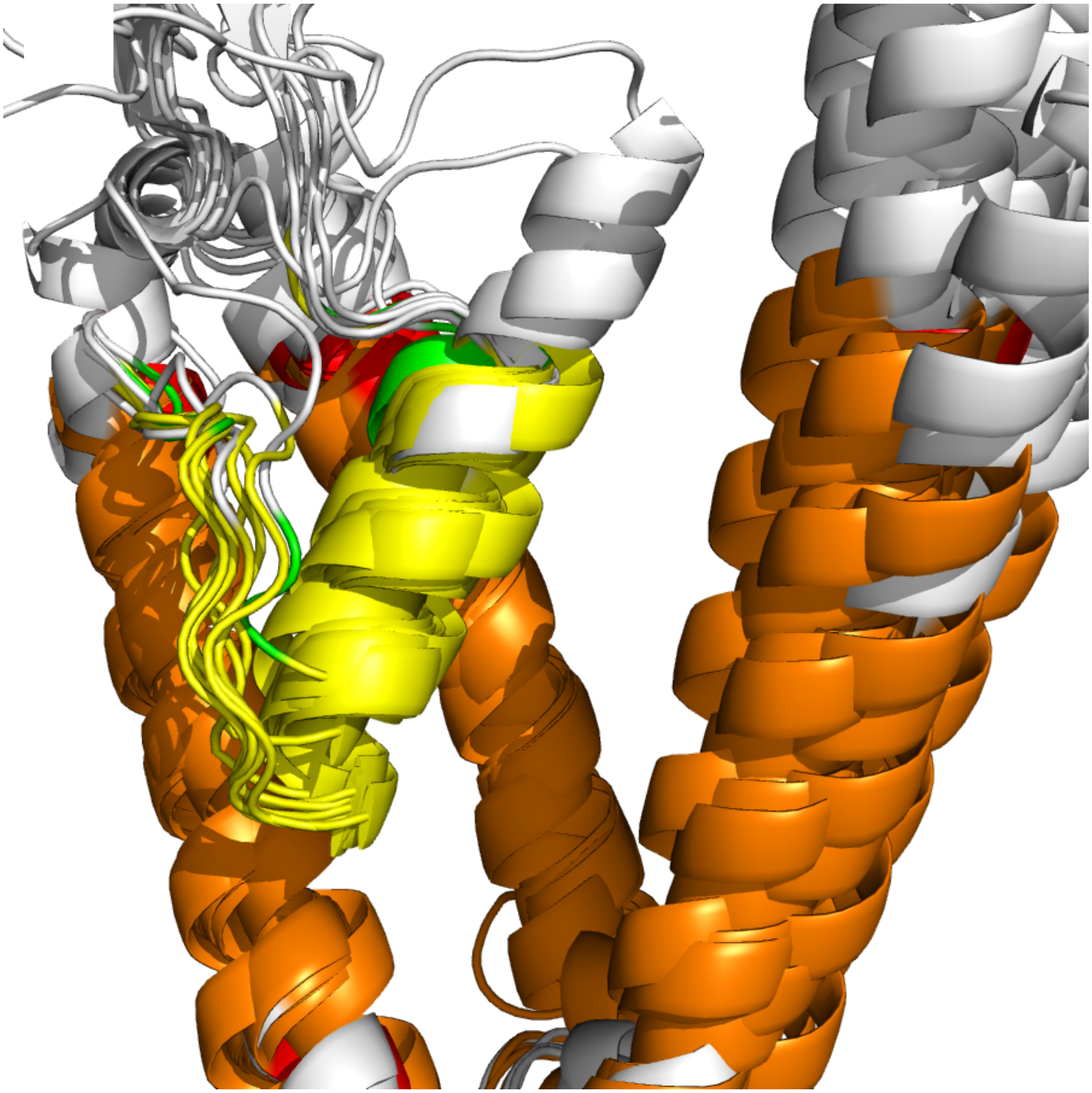

Figure S5. Automatic extension of TMSs and reentrant loops (RLs). Direct TmAlphaFold (tmAF) assignments are shown in orange (TMSs) and yellow (RLs). Extensions of TMSs and RLs by our approach (see Methods) are shown in red (TMSs) and green (RLs). Regions not involved in tmAF assignments are shown in gray. Obvious tmAF mispredictions were ignored (e.g., TMSs assigned as reentrant loops). All proteins in this figure belong to family GIC (TC: 1.A.10): A0A077YZQ7, A0A077Z8K1, A0A0K0J2U4, A0A0N4UQF3, A0A0R4ID43, A0A1D6GTI9, A0A1D6GTJ2, A0A1D6HQP8, A0A286Y9Q6, A0A2R8Q9Y5, A0A2R8QDR4, A0A3P7ET42, A0A3P7GM64, E7F5R2, G5EGQ9, I1J8B7, I1KFC6, I1M634, K7LWX1, O01623, P19492, Q01812, Q0KI42, Q0KIF2, Q10914, Q13002, Q8BMF5, Q8GXJ4, Q8MXV8, Q91756, Q9TVI0, and Q9VDH5.
