# Supplementary material for: The Pentameric Ligand-Gated Ion Channel Family: A New Member of the Voltage Gated Ion Channel Superfamily?
Source: Int J Mol Sci. 2024 May 3;25(9):5005. doi: 10.3390/ijms25095005 (PMC11084639; doi:10.3390/ijms25095005)
Supplement: Supplementary file 1 [file ijms-25-05005-s001.zip › Table_S1.pdf]

Table S1: Top pLIC vs VPC 3D structural alignments. Data are organized in 5 columns: (1) PDB IDs, including the chain, of the pLIC and VPC structures being compared; (2) Root Main Square Deviation (RMSD) score; (3) TM-score; (4) length of the structures being compared. The large hydrophilic loop between TMSs 3 and 4 was removed to focus the alignments of the TMSs. (5) alignment coverage was calculated based on residues within the membrane plane. Rows are sorted by TM-score. See Methods for details.

Alignment (pLIC vs VPC)	RMSD (Å)	TM-score	Length	Coverage
2MAW_A vs 4G7V_S	3.66	0.55317	100	91.4%
2MAW_A vs 4G7Y_S	3.63	0.51823	100	84.8%
2LM2_A vs 4G7V_S	3.91	0.51119	103	83.2%
6PV7_A vs 4G7V_S	3.92	0.51022	105	79.1%
2LM2_A vs 4G7Y_S	3.88	0.50932	100	83.8%
6CNJ_A vs 4G7Y_S	3.98	0.50499	103	82.2%
