# Supplementary material for: The Pentameric Ligand-Gated Ion Channel Family: A New Member of the Voltage Gated Ion Channel Superfamily?
Source: Int J Mol Sci. 2024 May 3;25(9):5005. doi: 10.3390/ijms25095005 (PMC11084639; doi:10.3390/ijms25095005)
Supplement: Supplementary file 1 [file ijms-25-05005-s001.zip › Table_S2.pdf]

Table S2. Top pLIC vs GIC 3D structural alignments. Data are organized in 5 columns: (1) PDB IDs, including the chain, of the LIC and GIC structures being compared; (2) Root Main Square Deviation (RMSD) score; (3) TM-score; (4) length of the structures being compared. The large hydrophilic loop between TMSs 3 and 4 was removed to focus the alignments of the TMSs. (5) alignment coverage. Rows are sorted by TM-score. See Methods for details.

Alignment (pLIC vs GIC)	RMSD (Å)	TM-score	Length	Coverage
1OED_C vs 6NJN_A	4.06	0.42547	78	61.42%
1OED_E vs 6NJL_B	3.46	0.42339	71	55.47%
1OED_C vs 5VOU_A	3.89	0.42155	77	60.63%
1OED_C vs 6NJN_C	3.97	0.42153	77	60.63%
1OED_C vs 5VOT_B	3.98	0.42106	74	58.27%
1OED_E vs 6NJM_B	3.48	0.42068	71	55.47%
1OED_C vs 6NJM_A	4.05	0.42032	77	60.63%
