# Supplementary material for: The Pentameric Ligand-Gated Ion Channel Family: A New Member of the Voltage Gated Ion Channel Superfamily?
Source: Int J Mol Sci. 2024 May 3;25(9):5005. doi: 10.3390/ijms25095005 (PMC11084639; doi:10.3390/ijms25095005)
Supplement: Supplementary file 1 [file ijms-25-05005-s001.zip › Table_S3.pdf]

Table S3. TMS and Reentrant Loop Statistics across the VIC superfamily. See Methods for details on structure retrieval from TmAlphaFold, clustering, mapping to TC identifiers, manual inspection and calculations. The table presents 8 columns: (1) Type of secondary structure elements composing the reentrant loops (RL), where H: Helix and L: Loop; (2) clusters were named with the TC identifier mapping to at least 60% of the structures in the cluster, with the additional constraint that at least 60% of all the structures in our data set that mapped to this TC identifier belong to this cluster; (3) size of the cluster in number of structures. Clusters with only one protein were ignored; (4) number of structures that were sampled for manual inspection; (5) mean and Standard Deviation (SD) of the number of TMSs; (6) mean and SD of TMS lengths; (7) mean and SD of RL counts; and (8) mean and SD of RL lengths. Note the similarity in multiple clusters between the average lengths of TMS and RLs.

Type	Cluster	Size	Sample	TMS number (±SD)	TMS lengths (±SD)	RL number (±SD)	RL lengths (±SD)
H-L	1_A_10	40	30	3.03 (±0.18)	19.84 (±3.23)	1.0 (±0.0)	20.93 (±3.24)
H-L	1_A_1_1	2	2	3.00 (0.0)	21.00 (±2.94)	1.0 (±0.0)	21.0 (±0.0)
H-L	1_A_1_13_1	3	3	4.67 (±1.89)	16.21 (±1.15)	1.0 (±0.0)	17.67 (±2.49)
H-L	1_A_1_2	40	30	5.90 (±0.30)	20.33 (±4.08)	1.0 (±0.0)	15.55 (±3.86)
H-L	1_A_1_29	2	2	3.00 (±0.0)	22.67 (±0.94)	1.0 (±0.0)	15.0 (±0.0)
H-L	1_A_1_4	5	5	5.40 (±0.80)	24.11 (±5.66)	1.2 (±0.4)	23.83 (±2.61)
H-L	1_A_1_5	20	20	6.00 (±0.0)	20.68 (±3.55)	1.0 (±0.0)	18.63 (±3.87)
H-L	1_A_1_7	9	9	6.00 (±2.31)	22.57 (±3.10)	1.78 (±0.42)	21.13 (±4.48)
H-L	1_A_2	64	30	2.00 (±0.0)	19.58 (±3.18)	1.0 (±0.0)	22.1 (±4.4)
H-H*	1_A_4	14	14	5.79 (±0.41)	21.09 (±5.19)	1.0 (±0.0)	15.5 (±3.02)
H-LH$^{\Psi}$	1_A_4_6	2	2	5.50 (±0.50)	22.64 (±6.80)	1.0 (±0.0)	16.5 (±2.5)
H-L	1_A_9_6_3	2	2	2.50 (±1.50)	24.20 (±2.48)	2.0 (±0.0)	15.5 (±0.5)
H-L	2_A_38	14	14	9.07 (±1.03)	19.00 (±3.87)	5.0 (±0.0)	18.24 (±3.7)
H-L	2_A_38_3	7	7	8.14 (±0.35)	20.84 (±3.64)	4.0 (±0.0)	18.89 (±3.96)

* Both helices are within the membrane plane.

$^{\Psi}$ It is not clear whether the second helix is in the membrane. Thus, it may be an H-L configuration.
