# Supplementary material for: The Pentameric Ligand-Gated Ion Channel Family: A New Member of the Voltage Gated Ion Channel Superfamily?
Source: Int J Mol Sci. 2024 May 3;25(9):5005. doi: 10.3390/ijms25095005 (PMC11084639; doi:10.3390/ijms25095005)
Supplement: Supplementary file 1 [file ijms-25-05005-s001.zip › Table_S4.pdf]

Table S4. Signature Regions (SR) recovery rates. Data are presented for families pLIC (TC: 1.A.9), GIC (TC:1.A.10) and VPC (TC: 1.A.51). MAST was run using a threshold E-value of $1x10^{-3}$ (see Methods).

Family	Number of homologs	Homologs with SR	Recovery rate (%)
pLIC	14,502	5,533	38.15
GIC	9,547	7,712	80.77
VPC	718	568	79.1
